# Three-dimensional analysis of incisive canals in human dentulous and edentulous maxillary bones

**DOI:** 10.1186/s40729-015-0012-4

**Published:** 2015-05-01

**Authors:** Masayuki Fukuda, Satoru Matsunaga, Kento Odaka, Yuuya Oomine, Masaaki Kasahara, Masahito Yamamoto, Shinichi Abe

**Affiliations:** Department of Anatomy, Tokyo Dental College, 2-9-18, Misaki-cho, Chiyoda-ku, Tokyo 101-0061 Japan

**Keywords:** Anatomy, Dental implant, Incisive canal, Maxilla, Micro-computed tomography, Three-dimensional structure

## Abstract

**Background:**

The purpose of this study was to reveal the structural properties that need to be considered in dental implant treatment by investigating differences between dentulous and edentulous maxillae in the three-dimensional (3D) microstructure of the incisive canals (ICs) and their surrounding bone.

**Methods:**

A total of 40 maxillary bones comprising 20 dentulous maxillae and 20 edentulous maxillae were imaged by micro-CT for 3D observation and measurement of the IC and alveolar bone in the anterior region of the IC.

**Results:**

The Y-morphology canal was most frequently observed at 60% in dentulous maxilla and 55% in edentulous maxilla. There was a significant difference between dentulous and edentulous maxillae in IC diameter and volume, but no significant difference between the two in the major axis of the ICs.

**Conclusions:**

The anatomic structure surrounding the IC has limited area for implant placement. Therefore, where esthetic and long-term maintenance requirements are taken into account, careful attention is needed when setting the placement position. Also, due to the resorption of bone, edentulous maxillae have a different IC morphology from dentulous maxillae, and therefore, a cautious approach is required.

## Background

In recent years, with the rapid spread of dental implant treatment and a dramatic increase in accidents, the importance of precise 3D data on the human jaw is ever-increasing [1-3]. Bone resorption after tooth extraction is an unavoidable event, and the morphological features of the jawbone change greatly after tooth loss [4-7]. Moreover, the maxilla has a thinner cortical bone than the mandible and presents with a structure where cancellous bone predominates [8]. Therefore, implants have a lower survival rate than the mandible, and the search for sites suitable for implant placement has become an urgent need [9]. The position of the incisive canal (IC) is one of the most important factors for implant placement in the premaxillary region, not only in terms of survival rate but also from an esthetic point of view [10-17]. The incisive canals tend to increase in size with aging and after tooth extraction [18]. If there is a possibility of perforating the incisive canals during implant placement, then a cortical bone/cancellous bone block graft can be adapted to the incisive fossa without removing the contents of the fossa. The contents are pushed posteriorly, thus allowing proper implant placement [19].

The ICs are located in the maxillary midline posterior to the maxillary central incisors, and through them descend the nasopalatine nerve and nasopalatine artery, so they are anatomically important structures [20,21]. Mardinger et al. classified IC morphology as observed from sagittal sections into four types: cylinder-shaped, funnel-shaped, hourglass-shaped, and banana-shaped [18]. Song et al. confirmed one to four branches at the midpoint of the length of the IC and made classifications depending on the number of branches. They reported that the major type of canal shape for the IC is Y-morphology, with four other types - spindle-shaped, very large, cystic, and narrowed - existing as minor types [22]. Tolga et al. ran 2D bone measurements on the IC and surrounding bone of the IC and observed the differences between dentulous and edentulous maxillary bone [23]. Accumulating more finely detailed data directly connected to clinical practice necessitates 3D structure analysis of the bone surrounding the IC.

Kim et al. reported on the usefulness of micro-CT in analyzing the internal microstructure of bones [24]. 3D analysis using micro-CT, which has an imaging resolution of 5 μm at the maximum, makes it possible to reveal very complex structural properties, including even the trabecular bone.

The purpose of the present study is therefore to reveal the structural properties that need to be considered in dental implant treatment by investigating the differences in the IC and their surrounding bone structures between dentulous and edentulous maxillary bones.

## Methods

### Specimens

 A total of 20 dentulous human maxillae and 20 edentulous maxillae samples were resected from dried adult skull bones obtained from the Department of Anatomy, Tokyo Dental College. Dentulous jaw samples with maxillary central incisors present on both sides and no midline diastema were selected to ensure they had no tooth loss.

**Figure 1:**
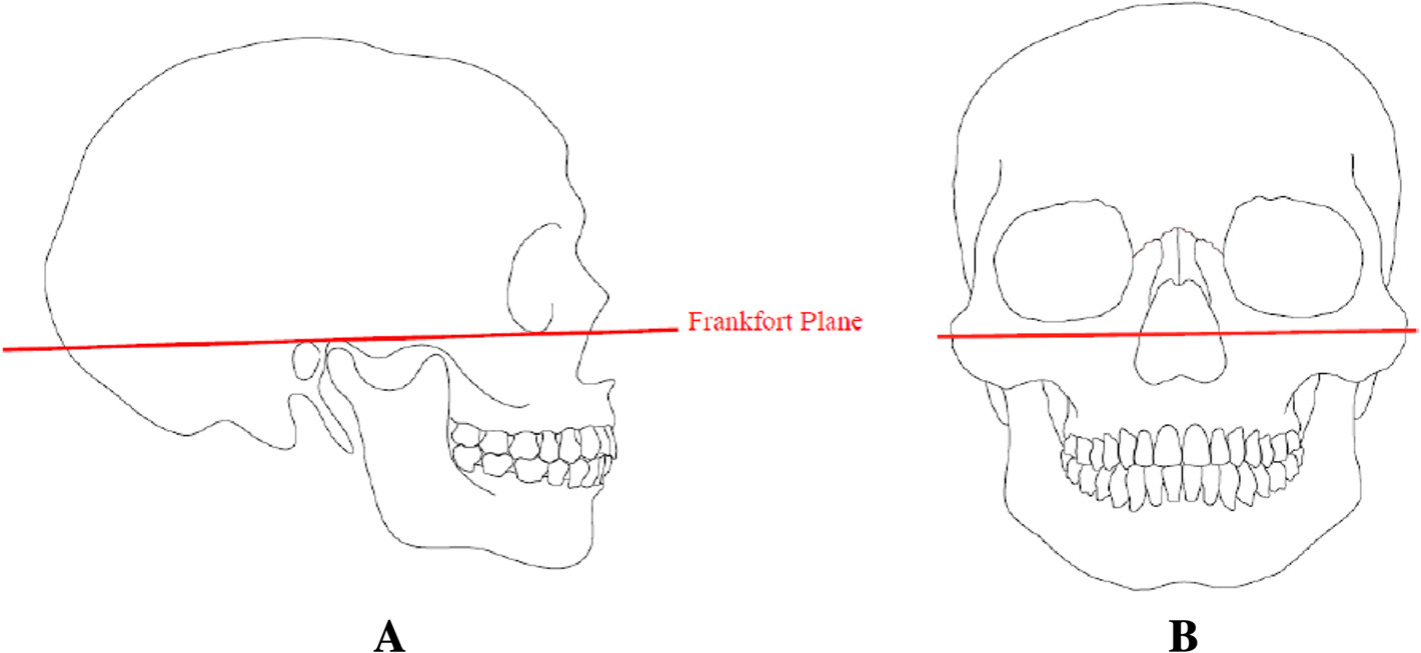
Method of collecting samples. **(A)** The skull was secured to a device and the Frankfort plane served as a reference. **(B)** A cut was made at the location of the zygomatic bone center, parallel to the Frankfort plane, and the maxillary bone was detached.

The skull was secured to a device, and the Frankfort plane was marked with India ink; this served as the reference for cutting in parallel from the position of the zygomatic bone center to collect the sample (Figure 1).

### Micro-CT imaging

The samples were imaged with a micro-CT system (HMX-225 Actis4; TESCO, Tokyo, Japan). Imaging conditions were as follows: matrix size 512 × 512, tube voltage 120 kV, and tube current 80 μA. The micro-CT imaging intensifier (I.I.) had a 1-in. CCD camera with a 4-in., 16-bit 1,024 × 1,024 scanning line. A total of 1,200 images of raw data were output with this camera. On the basis of this raw data, a back projection method was used to produce 2D slice data.

### Region of interest

Measurement was performed by preparing three-dimensional constructed models and thereafter taking a sagittal section at the region with maximum IC diameter. The region of interest was the distance from the anterior nasal spine to the maxillary first premolar. Taken as a reference plane at the time of measurement, the virtual plane was set so as to be a plane connecting the points of the largest bulge of the left/right maxillary tuberosities and the point of the incisive foramen, parallel to the Frankfort plane.

### Image processing

 3D construction software (VG Studio; Volume Graphics, Heidelberg, Germany) was used to observe the internal structure of the bone by 3D construction with volume rendering from the slice data. At the same time, image processing software (Mimics; Materialise, Leuven, Belgium) was used to separate the IC and other structures, followed by reconstruction with the IC shown in red.

### Classification of IC morphologies and calculation of their incidences

**Figure 2:**
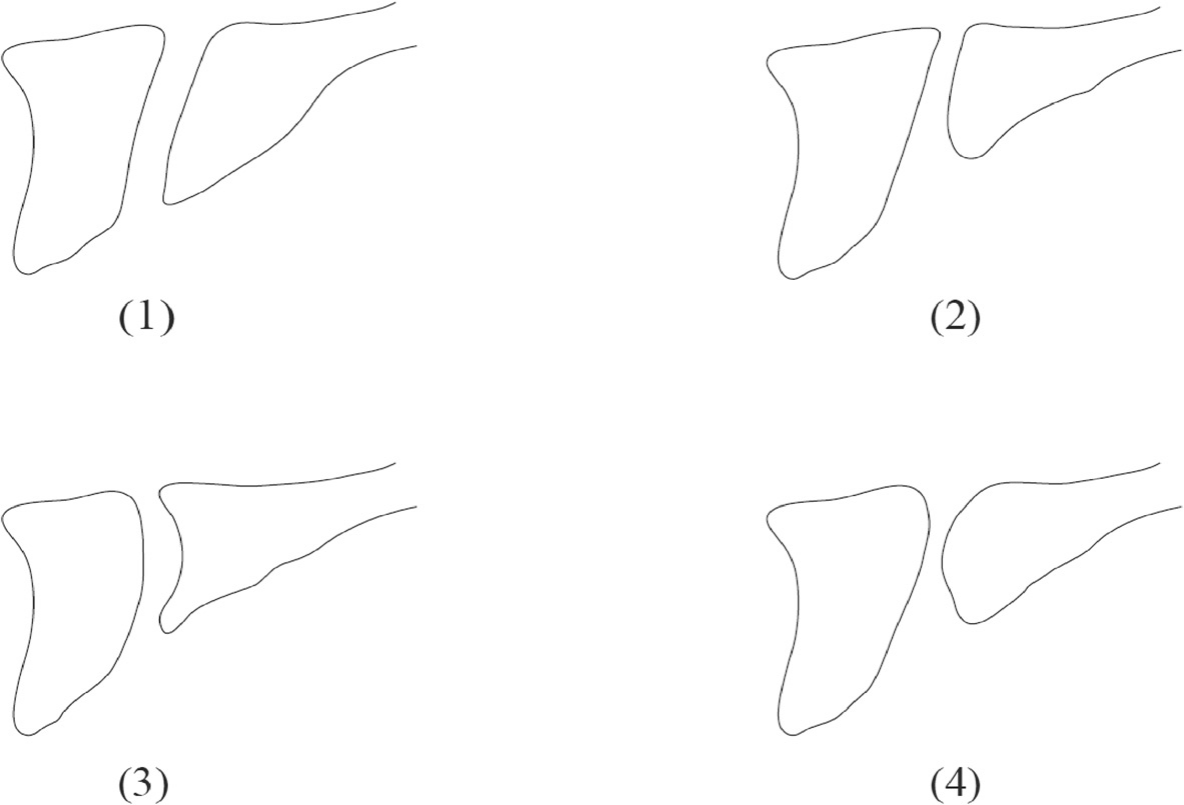
Morphological variations of IC as observed from the side [18]. **(1)** Cylinder-shaped. **(2)** Funnel-shaped. **(3)** Banana-shaped. **(4)** Hourglass-shaped.

As the principal item, those that divided completely in two by a bony wall following the nasal septum at a point approximately one fifth to the nasal side of the nasopalatine foramina were set as Y-morphology type, and those that did not were set as cylindrical type. As a secondary item, local IC morphologies in the sagittal cross section were taken as cylinder-shaped, funnel-shaped, banana-shaped, or hourglass-shaped, in accordance with Mardinger and Mraiwa classifications [18,25]. The incidence was calculated for both the principal item and the secondary item (Figure 2).

### Measurements of surrounding bone

**Figure 3:**
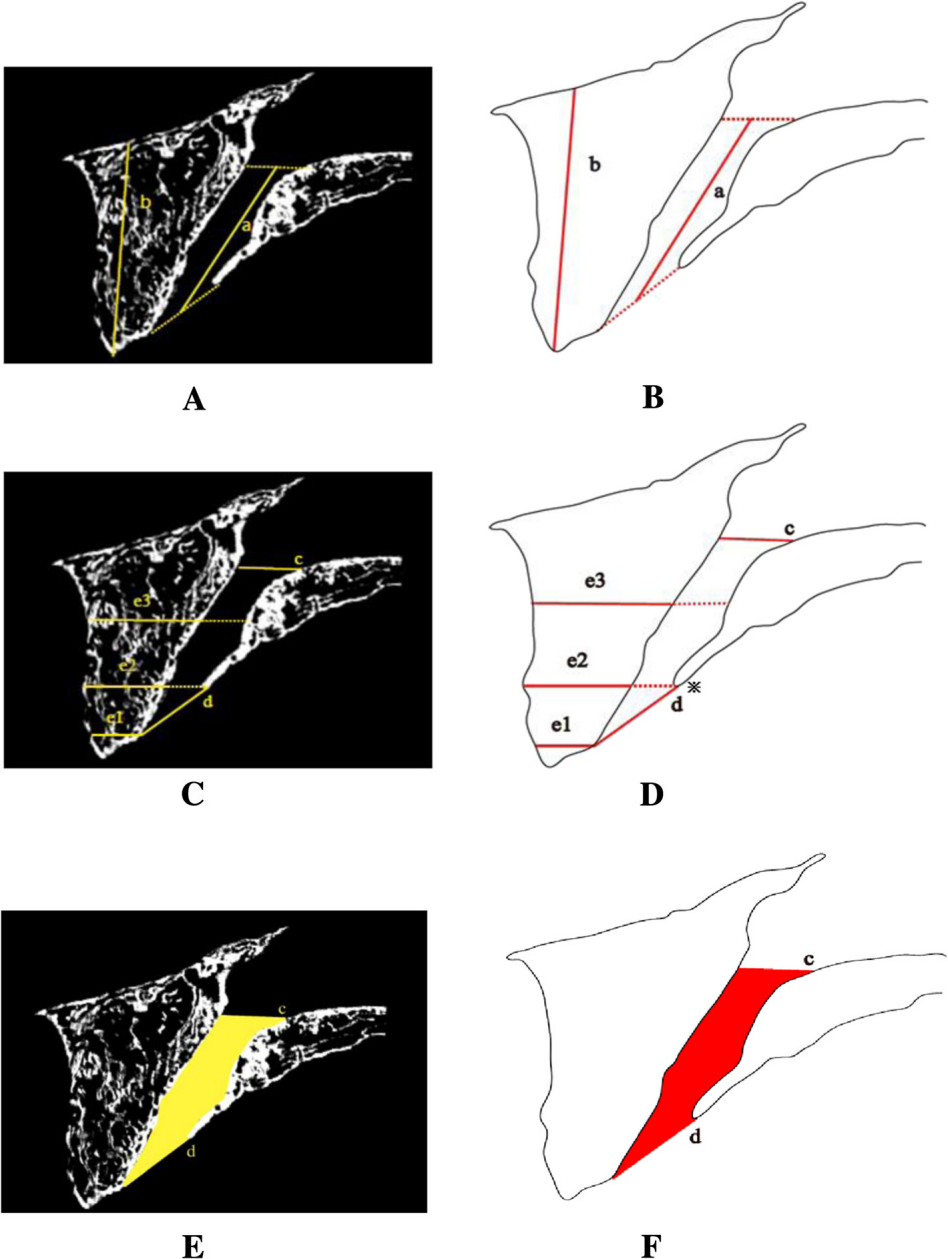
IC measurement items. **(A**, **C**, **E)** CT slice images observed from the side, with sagittal sections of the IC at the middle. **(B**, **D**, **F)** Schematic diagrams of A, C, and E. a: IC length, distance between the midpoint of c and midpoint of d. b: Height of the anterior alveolar bone of the IC, distance to the alveolar crest from a line passing through the anterior nasal spine, in parallel with the virtual plane. c: Diameter of the nasopalatine foramina, IC diameter passing over a line connecting the anterior nasal spine and the floor of the nasal cavity, in parallel with the virtual plane. d: Diameter of the incisive foramen, maximum distance from the point (asterisk) where the superoposterior angle changes significantly. e: Width of the IC anterior alveolar bone, distance of the anterior alveolar bone parallel to the virtual plane. (e1: position of the anteroinferior point matching the diameter of the incisive foramen; e2: position of the intermediate part (asterisk); e3: position of 50% of the major axis of the upper IC). f: Total volume from c to d.

Measurement software (TRI-3D/BON; Ratoc System Engineering, Tokyo, Japan) was used for the following bone measurements: IC length (a), IC anterior alveolar bone height (b), IC diameter (c, d), bone width of the IC anterior alveolar bone (e1, e2, e3), and IC volume (f) (Figure 3).

### Statistical analysis

 PASW Statistics 18 for Windows (SPSS Inc., Tokyo, Japan) was used for statistical processing. Student’s *t*-test was used to test for comparison between dentulous and edentulous maxillary bones. The *p* value was set to <0.05.

## Results

**Figure 4:**
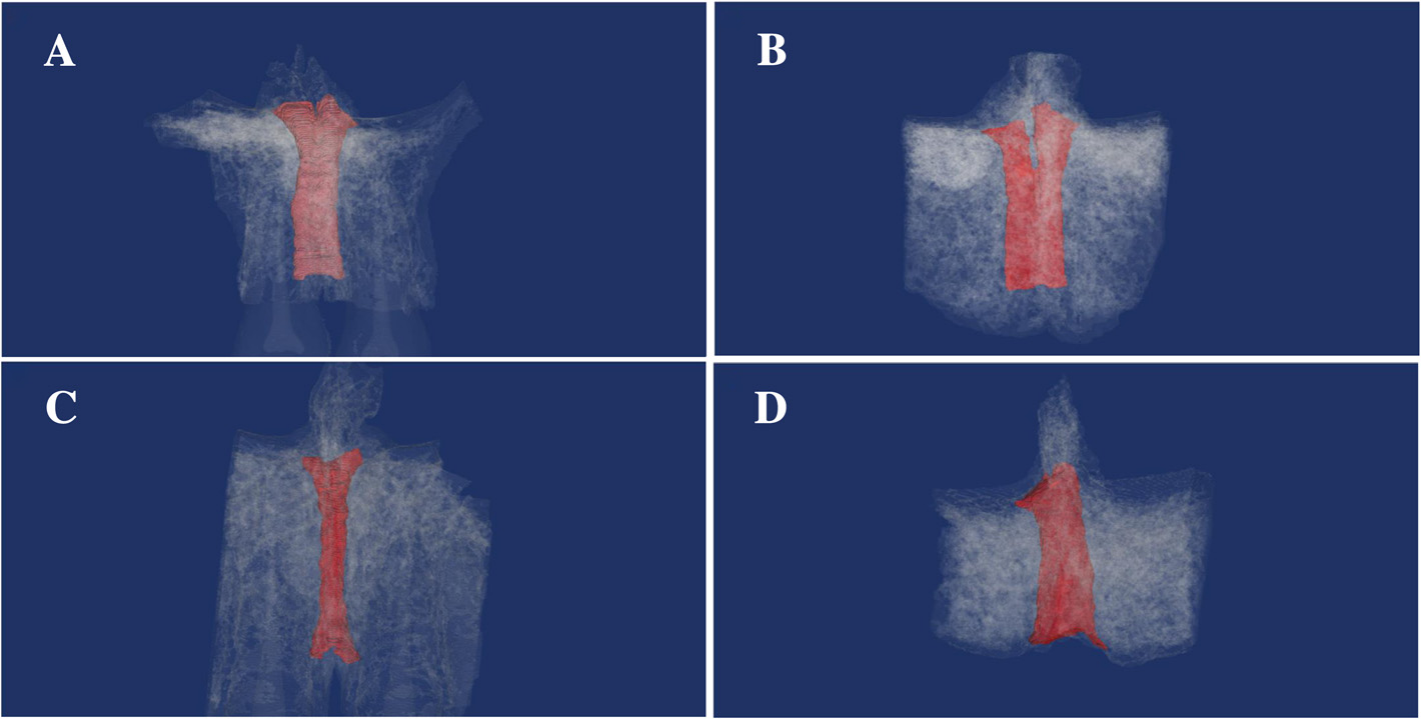
3D image constructions of the IC in dentulous maxilla and edentulous maxilla. After imaging by micro-CT, the slice data was used for 3D construction, with the IC shown in red. IC morphology as observed from the front view was classified into Y-morphology and cylindrical [22]. **(A)** Y-morphology of dentulous maxilla. **(B)** Y-morphology of edentulous maxilla. **(C)** Cylindrical of dentulous maxilla. **(D)** Cylindrical of edentulous maxilla.

**Figure 5:**
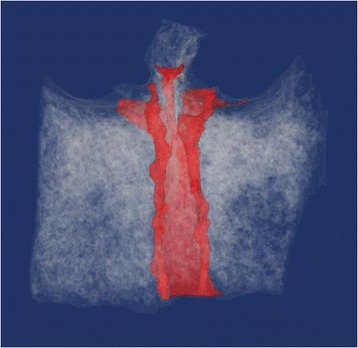
3D image constructions of IC showing a unique morphology. IC morphology with three or four branches was observed in one sample.

 Overall, 58% of all samples presented with the Y-morphology, divided completely in two by a bony wall following the nasal septum at a point approximately one fifth to the nasal side of the IC length, with two nasopalatine foramina opening on the floor of the nasal cavity. The incidence of each classification in the dentulous maxilla was as follows: 60% Y-morphology and 35% cylindrical. The corre sponding values in the edentulous maxilla were 55% and 25%. Next, the shape of the IC from the sagittal cross section was most often funnel-shaped for both dentulous and edentulous maxilla, at 50% and 45%, respectively. The cylinder-shaped was observed most frequently after the funnel-shaped morphology. Otherwise, 7.5% overall had two to four branches observed at the middle point of the length of the IC (Tables 1 and 2) (Figures 4 and 5).

**Table 1:** **Number of individuals and proportions based on classification of the principal item**

		**Classification of the IC from the overall appearance**	
		**Y-morphology**	**Cylindrical**
Dentulous	*n*	12	7
	%	60	35
Edentulous	*n*	11	5
	%	55	25
Dentulous + edentulous	*n*	23	12
	%	58	30

**Table 2:** **Number of individuals and proportions based on classification of the secondary item**

		**Classification of the IC from the sagittal cross section**			
		**Cylinder-shaped**	**Funnel-shaped**	**Banana-shaped**	**Hourglass-shaped**
Dentulous	*n*	9	10	0	1
	%	45	50	0	5
Edentulous	*n*	9	9	1	1
	%	45	45	5	5

Morphologies of the classification match those in Figure 2.

### Observation of bone structure

**Figure 6:**
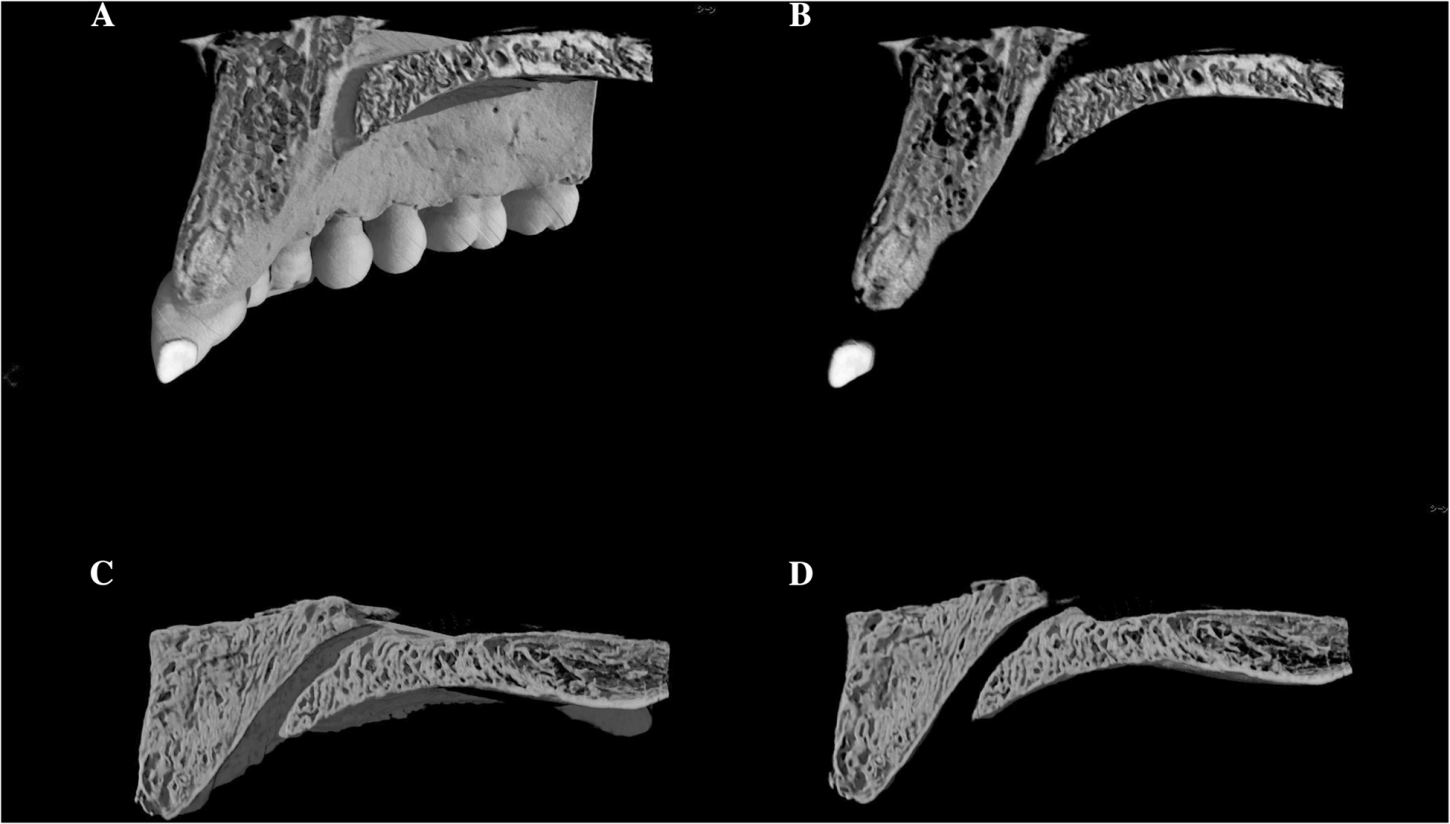
Representative images of bone internal structure of the bone surrounding the IC. In **(A-D)**, the 3D image constructions of dentulous maxilla and edentulous maxilla were cut in the labiopalatal direction and differences in properties pertaining to the structure of bone were observed. In (B) and (D), the samples were further sliced and observed in detail. (A, B) Relatively thick cortical bone; the cancellous bone travels in a direction parallel to the longitudinal axis of the tooth, and the density is low. (C, D) Cortical bone is especially thin in edentulous maxilla, and the cancellous bone travels with irregular arraying of fine trabecular bone. The density is very high.

Alveolar bone in the anterior region of the IC tended to be extremely thin on the cortical bone. In association with this, the trabecular bone structure of the alveolar process was such that in dentulous maxilla, the trabecular bone runs parallel to the longitudinal axis of the tooth and was distributed sparsely. In the edentulous maxilla, however, the arrangement of trabecular bone structure was irregular and very dense, representing a difference in bone structure characteristics (Figure 6).

### Descriptive analysis of the IC and alveolar bone

The mean IC length (a) was 11.75 mm for dentulous maxilla and 10.84 mm for edentulous maxilla. The mean alveolar bone height (b) anterior to the IC was 17.97 mm for dentulous maxilla and 14.01 mm for edentulous maxilla. The mean IC diameter in (c) showed results of 2.84 mm for dentulous maxilla and 3.56 mm for edentulous maxilla; in (d), it was 4.28 mm for dentulous maxilla and 5.40 mm for edentulous maxilla. The mean bone width for IC anterior alveolar bone was 5.80 mm for dentulous maxilla and 4.06 mm for edentulous maxilla at (e1), 5.61 mm for dentulous maxilla and 5.08 mm for edentulous maxilla at (e2), and 8.45 mm for dentulous maxilla and 8.42 mm for edentulous maxilla at (e3). The mean IC volume (f) was 60.67 mm^3^ for dentulous maxilla and 87.05 mm^3^ for edentulous maxilla (Table 3).

**Table 3:** **Comparative analysis of measurements items in dentulous maxilla and edentulous maxilla**

**Measurements**	**Dentulous (** ***n*** **= 20)**	**Edentulous (** ***n*** **= 20)**	***p***
	**Mean ± SD**	**Mean ± SD**	
a	11.75 ± 1.70 (mm)	10.84 ± 2.42	0.241
b	17.97 ± 3.16 (mm)	14.01 ± 3.50	0.001*
c	2.84 ± 0.79 (mm)	3.56 ± 0.91	0.022*
d	4.28 ± 1.49 (mm)	5.40 ± 1.44	0.020*
e1	5.80 ± 1.21 (mm)	4.06 ± 1.77	0.002*
e2	5.61 ± 1.13 (mm)	5.08 ± 1.87	0.358
e3	8.45 ± 1.84 (mm)	8.42 ± 1.59	0.972
f	60.67 ± 36.25 (mm^3^)	87.05 ± 32.34	0.036*

*p < 0.05*.* Measurements a to f match those in Figure 3.

## Discussion

Dental implant surgery in the premaxillary region often has limited bone for implant placement, and as a result, there is the risk of a poor prognosis in some instances [26]. In particular, the ICs are located in the anterior part of the hard palate, and the probability of adverse effects during implant placement in the premaxillary region is about 4% [27]. Therefore, practitioners must be aware of the risks of accidents and avoid them by using precise, detailed research data on the IC.

Close to 60% of all ICs exhibit Y-morphology. However, according to the report by Michael et al., the frequency of a cylindrical morphology of IC was 50% or more [28]. Otherwise, in terms of rare cases, Frederico et al. studied prospective implant patients using CBCT and reported that two ICs that are completely independent exist [29]. In the present study, instances where two or more IC branches existed accounted for 7.5% of the whole sample, and this suggests the possibility that they vary depending on various factors such as ethnicity or environment.

Regarding IC morphology as observed from the sagittal plane, on the other hand, funnel-shaped predominated, at 55% in dentulous maxilla and 50% in edentulous maxilla, followed by cylinder-shaped at 45% in dentulous maxilla and 45% in edentulous maxilla. This result is largely consistent with Mardinger et al.’s findings, namely, the principal shapes of the IC were shown to be funnel-shaped and cylinder-shaped, whether it be for dentulous or for edentulous maxilla. With funnel-shaped, expansion of the incisive foramen is observed, so depending on the angle of implant placement, there is a risk of perforating the IC and damaging the nasopalatine nerves and arteries. Next, the IC diameter (c, d) and volume (f) showed significant differences between dentulous and edentulous maxilla. This suggests that bone resorption due to the loss of teeth also has an impact on the IC. Bhola et al. reported that in order to obtain initial stability of the implant, there are two requisites: an implant fixture measuring 10 mm or longer must be used, and at the time of placement, there needs to be at least 3 mm or more alveolar bone from the implant body tip [30]. In the present study, where attention is limited to the alveolar bone height (b) in the anterior region of the IC, adequate bone for implant placement was present in 60% of edentulous maxilla. It is, however, desirable for there to be a thickness of 2 mm or more of hard tissue on the labial side (buccal side) of the implant, when the long-term prognosis is taken into account [31]. The mean bone width of the IC anterior alveolar bone was 4.05 mm in edentulous maxilla at e1. Considering the placement of an implant with a diameter of 4 mm, which is now commonly used, there are still comparatively many cases with high risk.

Typically, in implant surgery, a platform is prepared at the top of the alveolar bone [32]. However, there are reported cases where a slight gap created at the platform after placement became a space for bacteria to grow, resulting in about 1 to 2 mm of bone resorption due to inflammation of the peri-implant gingiva [33-35]. As gingival recession affects the esthetics, it seems possible that long-term prognosis could be affected by the fine cancellous bone and thin cortical bone observed in the present study.

## Conclusions

The structure surrounding the IC, anatomically, has limited area for implant placement, and when esthetic and long-term maintenance requirements are taken into account, careful attention is needed in setting the placement position. Also, due to the resorption of bone, edentulous maxillae have a different IC morphology from dentulous maxillae, and therefore, a cautious approach is required.

This study demonstrated that the IC displays many morphological as well as dimensional variations. These findings may assist clinicians in understanding the morphology and planning treatment.

## References

[1] Feldkamp LA, Goldstein SA, Parfitt AM, Jesion G, Kleerekoper M (1989). The direct examination of three-dimensional bone architecture in vitro by computed tomography. J Bone Miner Res.

[2] Ruegsegger P, Koller B, Muller R (1996). A microtomographic system for the nondestructive evaluation of bone architecture. Calcif Tissue Int.

[3] Sawada K, Nakahara K, Matsunaga S, Abe S, Ide Y (2013). Evaluation of cortical bone thickness and root proximity at maxillary interradicular sites for mini-implant placement. Clin Oral Implants Res.

[4] Atwood DA (2001). Some clinical factors related to rate of resorption of residual ridges. 1962. J Prosthet Dent.

[5] Atwood DA, Coy WA (1971). Clinical, cephalometric, and densitometric study of reduction of residual ridges. J Prosthet Dent.

[6] Atwood DA (1979). Bone loss of edentulous alveolar ridges. J Periodontol.

[7] Araújo MG, Lindhe J (2005). Dimensional ridge alterations following tooth extraction. An experimental study in the dog. J Clin Periodontol.

[8] Huang H, Richards M, Bedair T, Fields HW, Palomo JM, Johnston WM (2013). Effects of orthodontic treatment on human alveolar bone density distribution. Clin Oral Investig.

[9] van Steenberghe D (1989). A retrospective multicenter evaluation of the survival rate of osseointegrated fixtures supporting fixed partial prostheses in the treatment of partial edentulism. J Prosthet Dent.

[10] Saadoun AP, Sullivan DY, Krischek M, Le Gall M (1994). Single tooth implant–management for success. Pract Periodontics Aesthet Dent.

[11] Mecall RA, Rosenfeld AL (1991). Influence of residual ridge resorption patterns on implant fixture placement and tooth position. 1. Int J Periodontics Restorative Dent.

[12] Lazzara RJ (1994). Criteria for implant selection: surgical and prosthetic considerations. Pract Periodontics Aesthet Dent.

[13] Daftary F, Bahat O (1994). Prosthetically formulated natural aesthetics in implant prostheses. Pract Periodontics Aesthet Dent.

[14] Parel SM, Sullivan DY (1989). Esthetics and osseointegration.

[15] Rosenquist JB, Nystrom E (1992). Occlusion of the incisal canal with bone chips. A procedure to facilitate insertion of implants in the anterior maxilla. Int J Oral Maxillofac Surg.

[16] Scher EL (1994). Use of the incisive canal as a recipient site for root form implants: preliminary clinical reports. Implant Dent.

[17] Jacobs R, Lambrichts I, Liang X, Martens W, Mraiwa N, Adriaensens P (2007). Neurovascularization of the anterior jaw bones revisited using high-resolution magnetic resonance imaging. Oral Surg Oral Med Oral Pathol Oral Radiol Endod.

[18] Mardinger O, Namani-Sadan N, Chaushu G, Schwartz-Arad D (2008). Morphologic changes of the nasopalatine canal related to dental implantation: a radiologic study in different degrees of absorbed maxillae. J Periodontol.

[19] Artzi Z, Nemcovsky CE, Bitlitum I, Segal P (2000). Displacement of the incisive foramen in conjunction with implant placement in the anterior maxilla without jeopardizing vitality of nasopalatine nerve and vessels: a novel surgical approach. Clin Oral Implants Res.

[20] Standring S (2005). Gray’s anatomy.

[21] Rodella LF, Buffoli B, Labanca M, Rezzani R (2012). A review of the mandibular and maxillary nerve supplies and their clinical relevance. Arch Oral Biol.

[22] Song WC, Jo DI, Lee JY, Kim JN, Hur MS, Hu KS (2009). Microanatomy of the incisive canal using three-dimensional reconstruction of microCT images: an ex vivo study. Oral Surg Oral Med Oral Pathol Oral Radiol Endod.

[23] Tözüm TF, Güncü GN, Yildirim YD, Yilmaz HG, Galindo-Moreno P, Velasco-Torres M (2012). Evaluation of maxillary incisive canal characteristics related to dental implant treatment with computerized tomography: a clinical multicenter study. J Periodontol.

[24] Kim YJ, Henkin J. Micro-computed tomography assessment of human alveolar bone: bone density and three-dimensional micro-architecture. Clin Implant Dent Relat Res. 2013. doi:10.1111/cid.12109. 10.1111/cid.12109 23837565

[25] Mraiwa N, Jacobs R, Van Cleynenbreugel J, Sanderink G, Schutyser F, Suetens P (2004). The nasopalatine canal revisited using 2D and 3D CT imaging. Dentomaxillofac Radiol.

[26] Liang X, Jacobs R, Lambrichts I (2006). An assessment on spiral CT scan of the superior and inferior genial spinal foramina and canals. Surg Radiol Anat.

[27] Kraut RA, Boyden DK (1998). Location of incisive canal in relation to central incisor implants. Implant Dent.

[28] Bornstein MM, Balsiger R, Sendi P, Von Arx T (2011). Morphology of the nasopalatine canal and dental implant surgery: a radiographic analysis of 100 consecutive patients using limited cone-beam computed tomography. Clin Oral Implants Res.

[29] Neves FS, Oliveira LK, Ramos Mariz AC, Crusoé-Rebello I, De Oliveira-Santos C (2013). Rare anatomical variation related to the nasopalatine canal. Surg Radiol Anat.

[30] Bhola M, Neely AL, Kolhatkar S (2008). Immediate implant placement: clinical decisions, advantages, and disadvantages. J Prostho.

[31] Grunder U, Gracis S, Capelli M (2005). Influence of the 3-D bone-to-implant relationship on esthetics. Int J Periodontics Restorative Dent.

[32] Alonso-González R, Aloy-Prósper A, Peñarrocha-Oltra D, Peñarrocha-Diago MA, Peñarrocha-Diago M (2012). Marginal bone loss in relation to platform switching implant insertion depth: an update. J Clin Exp Dent.

[33] Esposito M, Ekestubbe A, Gröndahl K (1993). Radiological evaluation of marginal bone loss at tooth surfaces facing single Brånemark implants. Clin Oral Implants Res.

[34] Paul SJ, Jovanovic SA (1999). Anterior implant-supported reconstructions: a prosthetic challenge. Pract Proced Aesthet Dent.

[35] Saadoun AP, LeGall M, Touati B (1999). Selection and ideal tridimensional implant position for soft tissue aesthetics. Pract Proced Aesthet Dent.

